# Willingness to Pay for Elderly Telecare Service Using the Internet and Digital Terrestrial Broadcasting

**DOI:** 10.2196/ijmr.7461

**Published:** 2017-10-24

**Authors:** Satoshi Kaga, Teppei Suzuki, Katsuhiko Ogasawara

**Affiliations:** ^1^ Graduate School of Health Sciences Department of Health Sciences Hokkaido University Sapporo Japan; ^2^ Department of Health Sciences Faculty of Health Sciences Hokkaido University Sapporo Japan

**Keywords:** health services for the elderly, health information, remote consultation

## Abstract

**Background:**

In Japan over the past few years, more attention has been focused on unnoticed solitary death in the context of an aging society and the trend toward nuclear family. A number of institutions and companies have implemented a prevention measure with digital terrestrial broadcasting telecare services for the elderly: Hokkaido University; TV-Asahi Corporation; Hitachi, Ltd; Iwamizawa City; Hokkaido Television Broadcasting Co, Ltd; and Hamanasu Information Co, Ltd. Although this system is provided free of charge as a demonstration test, determining the appropriate price for the service is required for its sustainable operation.

**Objective:**

The aim of this study was to quantify individual willingness to pay (WTP) so as to test the tenability of digital terrestrial broadcasting service for elderly telecare.

**Methods:**

We used the contingent valuation method (CVM) to estimate the WTP for this service among 305 citizens (valid response rate 76.0%) living in Japan. A questionnaire survey was conducted for people aged 18 to 100 years according to Japanese age distribution from September 2016. To elicit WTP, we adopted a *double-bound dichotomous choice method* to ask the respondents whether they agree or disagree with the price we offered.

**Results:**

The median WTP for this service’s monthly fee is estimated to be 431 JPY (approximately US $3.7). The finding suggests that gender (0.66, *P*=.01), health consciousness (1.08, *P*=.01), willingness to use (2.38, *P*<.001), and seeing others less than once a week (1.00, *P*=.06) made a positive effect on WTP.

**Conclusions:**

We conclude that reliable WTP was elicited by CVM based on an Internet survey. Calculated median WTP for digital terrestrial broadcasting service for elderly telecare was 431 JPY (approximately US $3.7). In the analysis of factors that affect WTP, *constant factors*, *log-bid*, *health consciousness*, *gender*, *see others less than one time for week*, and *willingness to use* made positive effect to probability of acceptance. In comparison of WTP in different groups, age groups showed that WTP of the elderly group was higher than WTP of the middle age group and younger age group. However, WTP surveys need to be carefully conducted to minimize the sampling bias and allocate accurate structure of gender distribution.

## Introduction

### Aging Society

Japanese society has been known as an aging society. Coulmas defined the percentage of an aging society as follows: “There are three different types of societies based on the proportion of elderly. Aging society: 7-14% of the population are 65 years or older. Aged society: 14-21% of the population are 65 years or older. Hyper-aged society: 21% or more of the population are 65 years or older.” [1]. In 1970, the Japanese society was defined as an aging society, aged society in 1994, and in 2004, it became a hyper-aged society, and we expect this trend to continue [2]. Aging society comes with many social problems such as lack of medical resources and increase in social security expenses, and finding a solution to these problems has become an urgent task for Japan [3].

One of the biggest issues among aging societies is unnoticed isolated death. According to the Tokyo medical examiner’s office [4], unnoticed isolated death is defined as “death seen by no-one on their deathbed and mostly in home.” The reason for this social phenomenon is likely to be caused by the changing family structure, such as the nuclear family [5].

Although surveys about isolated elderly deaths in Japan have not been conducted, in Tokyo alone, 1973 males and 1143 elderly females were found after their death in their home, reported by Tokyo Medical Examiner’s Office. Moreover, 15,603 unnoticed isolated deaths were found in Japan more than 4 days after their death, reported by the Ministry of Internal Affairs and Communication [6]. The delay in finding of isolated death has become an increasing social cost [7]. The cost they need to pay is listed in Table 1.

**Table 1:** Social cost of unnoticed isolated death.

Payer		Price, JPY (US $)
**Family or guardian of elder (guarantor)**		
	Removing fluids and filth of decayed body	20,000-350,000 (173-3040)
	Elimination of pest	15,000-50,000 (129-434)
	Deodorize and disinfect	20,000-100,000 (173-868)
	Property value goes down	
	If it’s a public residence and has no guarantor for the room, all expenses above become public expenditure	
**Government**		
	Administrative autopsy	250,000-500,000 (2162-4325)
	Police (increase of work load)	
	Reregistration of individual record, cremation, cleaning out their belongings, etc	

Autopsy cost is mentioned as a public expenditure. However, this cost can be avoided if a death certificate is issued by the hospital, and thus, the early finding of disorder before death would save government expenses. The government [8,9] and several companies have offered methods to solve this problem [10-13].

### Previous Research

In Iwamizawa City, Hokkaido, Japan, in cooperation with Hokkaido University; TV-Asahi Corporation; Hitachi, Ltd; Iwamizawa City; Hokkaido Television Broadcasting Co, Ltd; and Hamanasu Information Co, Ltd, have been conducting demonstration experiments of elderly telecare services using the Internet and digital terrestrial broadcasting to enhance health consciousness of the elderly and to improve elderly telecare [14].

**Figure 1:**
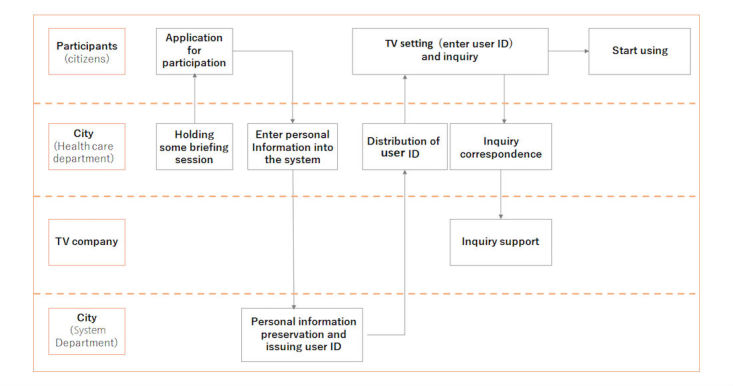
Operational flow to start.

About participation in this project, we connected the Internet to the television at the homes of participants who gave agreement. After issuing the participant’s user ID and password on the dedicated system, we made an Internet connection of the participant’s television and personal ID setting to their television. Registered TV in this study refers to a television that is ready to receive telecare services after personal ID setting is completed. The initial setting operation flow is shown in Figure 1. The person in charge of the city went for the equipment setting at the elderly home.

**Figure 2:**
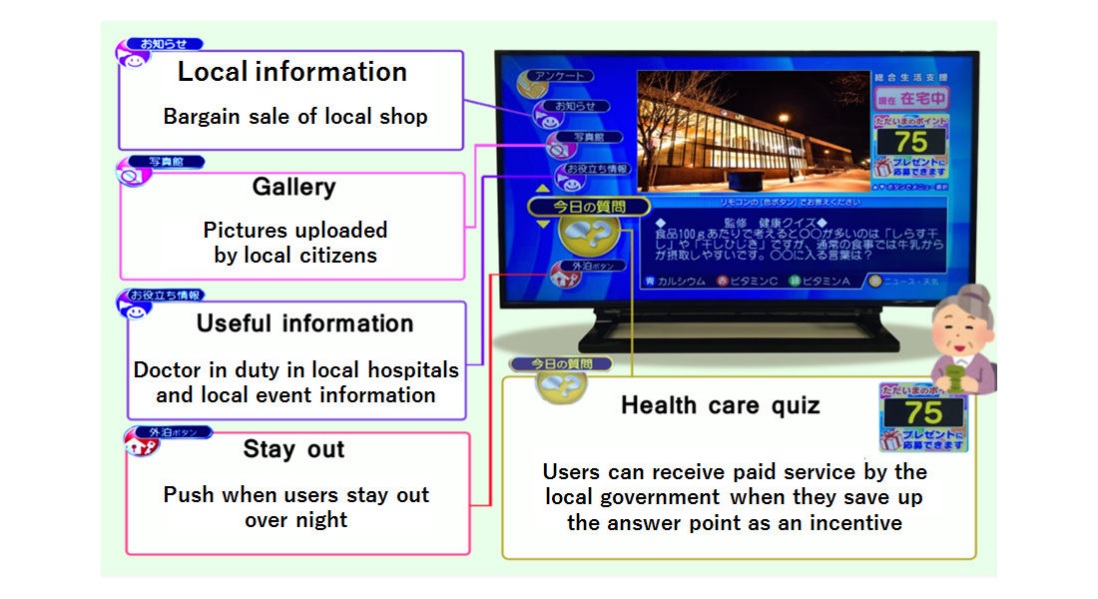
Contents of elderly telecare service.

**Figure 3:**
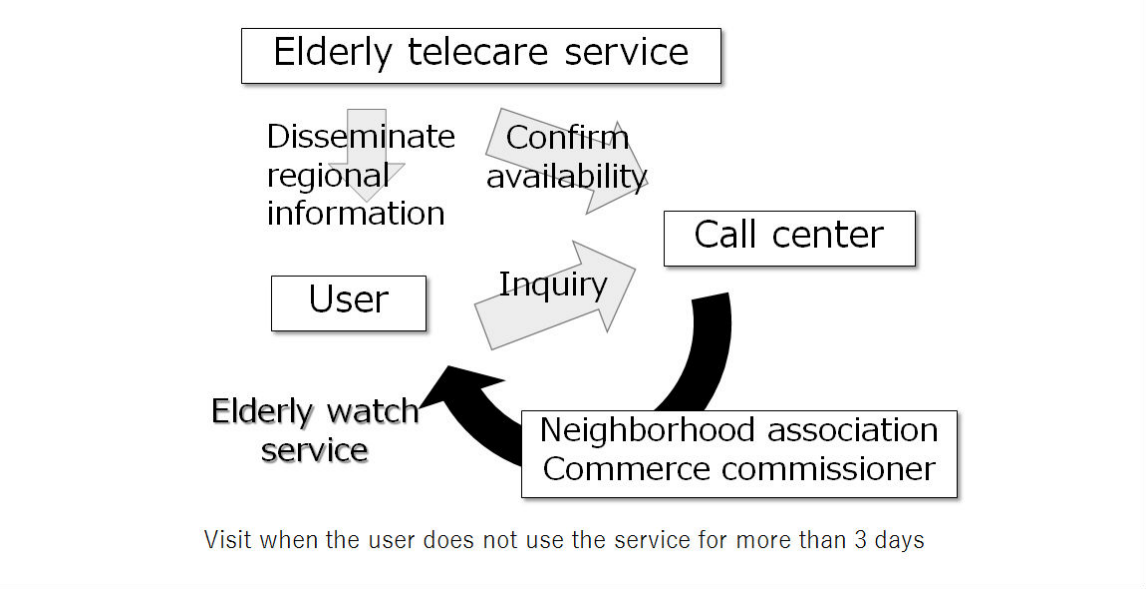
Elderly telecare service.

In this service, when a registered TV is not used for 3 days, the call center will make a confirmatory telephone call to the registered individual, and if the individual cannot be reached, then the neighborhood association and civil servant committee will visit the registered location to make sure the individual is not in any fatal situation. Other than the elderly telecare function, this service also offers information about health care, local events, and doctors on duty in local hospitals (Figures 2 and 3).

Although this system has been provided free of charge as a demonstration test, obtaining a certain level of return of economic investment is required for the sustainable operation of this service. The aim of this study was to quantify individual willingness to pay (WTP) measures of a digital terrestrial broadcasting service for elderly telecare.

## Methods

### Contingent Valuation Method

In this study, we used the contingent valuation method (CVM) to quantify individual WTP, which is widely used in economic evaluations [15], for example, in the health, transportation, and environment sectors. The CVM survey is used to elicit WTP values for a hypothetical change in the availability of nonmarket goods or services. Individual WTP values are generally elicited through dichotomous choice. The procedure is to ask a random sample of individuals via an Internet survey if they would be willing to pay a certain amount for a hypothetical change in the availability of a particular service. The test samples will be given two yes or no questions regarding price (Table 2). The second price is lower for people answering *no* and higher for people answering *yes* [16].

**Table 2:** Question price patterns in JPY (US $).

Price-first question, JPY(US $)	Upper price-second question (first question: yes)	Lower price-second question (first question: no)
500 (4.3)	750 (6.5)	250 (2.1)
1000 (8.6)	1500 (13.0)	500 (4.3)
2000 (17.3)	2500 (21.7)	1500 (13.0)
3000 (25.9)	3500 (30.4)	2500 (21.7)

### Data Collection

We decided to conduct an Internet survey because it is more cost-efficient and faster than mail or face-to-face interviews. A total of 400 Japanese citizens ranging from 18 to 100 years of age were chosen to participate in this survey. However, to reflect the Japanese population [17], the sample was strategically selected according to the Japanese population structure and then divided into 10 groups according to the subjects’ age and residential area. We expected WTP of people living in urban cities to be higher than that of people in rural areas because unnoticed isolated death in urban cities is regarded as a problem reported by the Ministry of Internal Affairs and Communications [18], and age can also affect WTP. For a definition of age distribution, refer to Table 3. For a definition of population distribution in urban areas, refer to Table 4.

**Table 3:** Definitions of age distribution.

Definitions	Age (years)	Description
Younger	18-39	Low WTP^a^ for service because they have no experience or need for this service
Middle	40-65	High WTP for service because this generation is mostly like to have parents older than 65 years, and so, they are willing to pay more
Elderly	Over 65	Potential users of this service who are older than 65 years can have a higher WTP because demand is relatively higher than the other age groups

^a^WTP: willingness to pay.

**Table 4:** Definition of population distribution in urban areas.

Urban area	City name	Population	Area (km²)	Population density (people/km²)
Sapporo area	Sapporo	2,584,880	4514	573
Sendai area	Sendai	2,169,757	5970	363
Kanto area	Saitama, Chiba, Tokyo, Yokohama, Kawasaki, and Sagamihara	36,923,193	14,034	2631
Niigata area	Niigata	1,421,694	5345	266
Shizuoka and Hamamatsu area	Shizuoka, Hamamatsu	2,741,028	4982	550
Chukyo area	Nagoya	9,107,414	7072	1288
Kinki area	Kyoto, Osaka, Sakai, Kobe	19,341,976	13,033	1484
Okayama area	Okayama	1,647,892	3637	453
Hiroshima area	Hiroshima	2,099,514	5048	416
Kita-Kyushu area, Fukuoka area	Kita-Kyushu, Fukuoka	5,515,427	5731	962
Utsunomiya area	Utsunomiya	1,086,898	5455	199
Matsuyama area	Matsuyama	717,687	2272	316
Kumamoto area	Kumamoto	1,476,435	4251	347
Kagoshima area	Kagoshima	1,152,748	3458	333

### WTP Estimation

**Figure 4:**
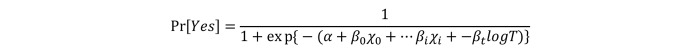
The model includes all variables.

We used a double-bound dichotomous logit model; median WTP is estimated as a representative value. Median indicates that 50.0% of the respondents agree with the offered amount [19,20]. This is important because our project is a public project [21], and median is usually the deciding factor for many public decisions. For estimating the WTP parameter, we decided to use a logit model because we expect WTP to stagnate after the price reaches a certain amount [22,23]. First, we estimate WTP with a model including all variables (Figure 4). Next, we estimate WTP with a model including variables with significant influence, and finally, we compare the WTP between different age groups and area groups with the Krinsky and Robb credential interval [24].

**Table 5:** Expected effects to willingness to pay (WTP).

Validation questions	Details
I agree to this project but I don’t think it’s worth paying.	Valid answer
I would like to use this service but I can’t afford it.	Valid answer
I could not decide with this information.	Invalid answer
Even if it’s free to use, I don’t think it’s worth paying.	Valid answer
Write your own reason if any ( ).	

**Table 6:** The questionnaire for excluding invalid answers.

Factor		Affect to WTP^a^
Age		Health risk goes up as people get older; therefore, WTP is likely to increase
**Gender**		
	Male	Not significant
	Female	
**Family structures**		
	Living alone	If respondents live alone, they would feel necessary to use telecare service; therefore, WTP is likely to increase
	Living with 2 or more people	
**Family net income**		
	More than 8 million JPY	The more income they have, the more money they can consume
	Less than 8 million JPY	
**Personally knows people who live alone**		
	Yes	If respondents have an acquaintance who lives alone, they would feel the necessity of a telecare service; therefore, WTP is likely to increase
	No	
**Health consciousness**		
	Yes	If respondents are health conscious, they could give this system a good reputation; therefore, WTP is likely to increase
	No	
**Willingness to use**		
	Yes	If respondents have willingness to use, they would agree to pay
	No	
**Concern toward health**		
	Yes	If respondents have anxiety regarding their health, they would feel the necessity of a telecare service
	No	
**See others less than once a week**		
	Yes	If respondents go a week without seeing anyone, they could be in a state of social isolation in the local community and consider the risk of dying alone
	No	

^a^WTP: willingness to pay.

### Questionnaire Design

Regarding questionnaire design (Table 5), we prepared questions to ask about basic properties, such as “family structure,” “family net income,” “Do you have anyone who lives alone around you?,” and questions related to factors affecting WTP such as “Do you habitually care about your health?,” “Would you like to use this service?,” “Are you concerned about your health?,” “Do you have weekly meetings with anybody?,” and “If this service costs JPY, would you be willing to pay? Think about the decrease in your income or saving,” and “How about if this service costs ~JPY? Would you be willing to pay?” We also prepared questionnaires for excluding invalid answers (Table 6).

## Results

### Results of Questionnaire

The response rate of this questionnaire was 100%, but 95 answers were excluded because of invalid answers. Therefore, 305 (76%; Tables 7-9) answers were analyzed.

**Figure 5:**
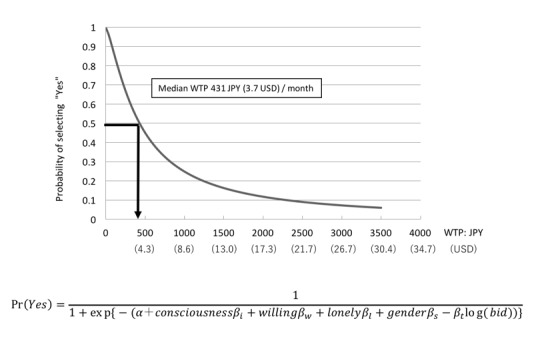
Estimated willingness to pay (WTP) model.

According to our initial calculation, the WTP average was calculated to be 1525 JPY (US $13.1). However, this figure is inaccurate, as it does not take into account the concern of overvaluation. As a general countermeasure, it is recommended to cut the hem with the maximum WTP [21]. In the second calculation, we decided to insert a maximum value of 3500 JPY (US $30.2), which gave us a new average of 809 JPY (US $6.9), and a median of 431 JPY (US $3.7; Figure 5, Table 10). In addition, we confirmed that multicollinearity between each variable does not occur, using the variance inflation factor (VIF; Table 11).

**Table 7:** Basic properties (305 valid responses).

Characteristics		Sample, n (%)
**Gender**		
	Male	191 (63)
	Female	114 (37)
**Age in years**		
	18-39	82 (27)
	40-64	123 (40)
	Over 65	100 (33)
**Family structure**		
	Living alone	67 (22)
	Living with spouse	97 (32)
	Living with spouse and child or children	106 (35)
	Single father or mother or living with a married child	3 (1)
	Three or more generations	21 (7)
	Others	11 (3)
**Family net income in JPY**		
	Lower than 2 million	51 (17)
	2-4 million	86 (28)
	4-6 million	71 (23)
	6-8 million	41 (13)
	8-10 million	33 (11)
	10+ million	23 (8)

**Table 8:** Factor summary.

Factors		Sample, n (%)
**Age in years**		
	18-39	82 (27)
	40-64	123(40)
	Over 65	100(33)
**Gender**		
	Male	191 (63)
	Female	114 (37)
**Family structures**		
	Living alone	67 (22)
	Living with someone	238 (78)
**Family net income in JPY**		
	More than 8 million	56 (18)
	Less than 8 million	249 (72)
**Personally knows people who live alone**		
	Yes	105 (34)
	No	200 (66)
**Health consciousness**		
	Yes	258 (84)
	No	47 (16)
**Willingness to use**		
	Yes	158 (51)
	No	147 (49)
**Concern toward health**		
	Yes	113 (37)
	No	192 (63)
**See others less than once a week**		
	Yes	19 (6)
	No	286 (94)

**Table 9:** Summary of price patterns.

Price in JPY (US $)	Yes-Yes	Yes-No	No-Yes	No-No
500 (4.3)	20	9	16	33
1000 (8.6)	16	13	17	35
2000 (17.3)	11	6	0	59
3000 (25.9)	10	5	4	51

**Table 10:** Estimated willingness to pay (WTP).

Willingness to pay	Price in JPY/month (US $)
Median	431 (3.7)
Average	1525 (13.1)
Average (truncated at the maximum bid)	809 (6.9)

**Table 11:** Estimated parameters of the willingness to pay (WTP) model.

Variables	Parameter (standard error)	*P* value	Variance inflation factor
Constant: alpha	5.312 (0.846)	<.001	
Log(bid)	−1.310 (0.121)	<.001	1.036
Health consciousness	1.086 (0.439)	.01	1.039
Gender	0.667 (0.272)	.01	1.004
Willingness to use	2.388 (0.303)	.001	1.036
See others less than once a week	1.003 (0.528)	.06	1.029

**Figure 6:**
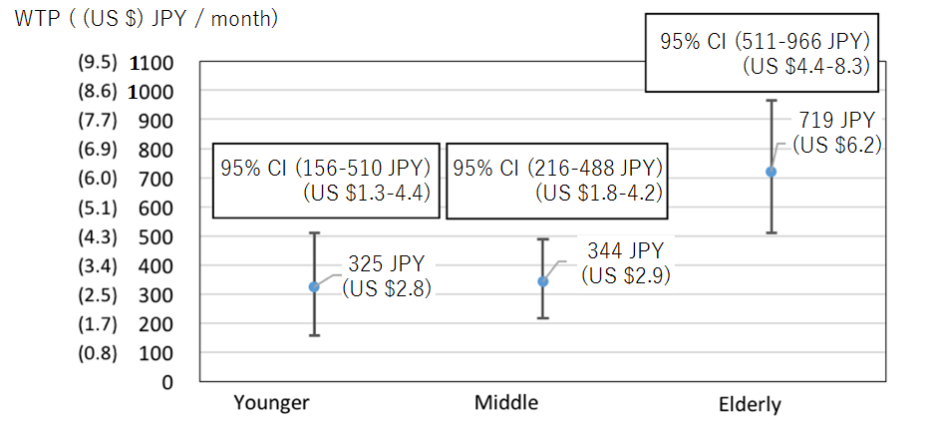
CI of willingness to pay (WTP) for different generations.

**Figure 7:**
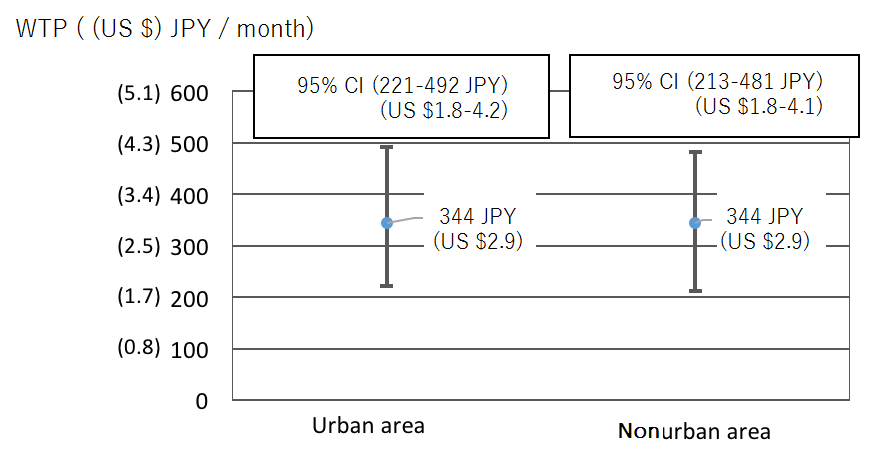
CI of willingness to pay (WTP) for urban and nonurban area.

If we look at the comparison between different generations (Figure 6), we can see that younger generations (18-39 years) and middle generations (39-64 years) do not have significant differences. However, the older generation (over 65 years), who are potential users in the future, showed a much higher WTP and is significantly different from the other generations. Although we expected that people of different locations would make a significant difference in the WTP (Figure 7), the result shows that there are no significant differences for people living in urban and nonurban areas.

### Analysis of Factors Affecting WTP

In analysis of factors affecting WTP, variables with significant changes will be isolated and analyzed in more detail (Tables 12 and 13). We adopted a logistic linear model for the following isolated factors: *constant factors*, *log-bid*, *gender*, *health consciousness*, *willingness to use*, and *not seen for over a week*.

**Table 12:** Analysis of all factors that affect willingness to pay (WTP).

Variables	Parameters	*P* value
Constant	5.346	<.001
log(bid)	−1.326	<.001
Gender	0.669	.02
Age	−0.003	.67
Living alone	0.281	.40
High income	0.187	.57
Having an acquaintance who lives alone	0.199	.47
Health consciousness	1.011	.03
Willingness to use	2.422	<.001
Anxiety to health	0.354	.20
See others less than once a week	0.852	.12

**Table 13:** Analysis with only isolated factors that affect willingness to pay (WTP).

Variables	Parameters	*P* value
Constant	5.312	.001
ln (offer amount)	−1.310	.001
Gender	0.667	.01
Interest in health	1.086	.01
Willingness to pay to service	2.388	.001
See others less than once a week	1.003	.06

## Discussion

### WTP With Relation to Price

**Figure 8:**
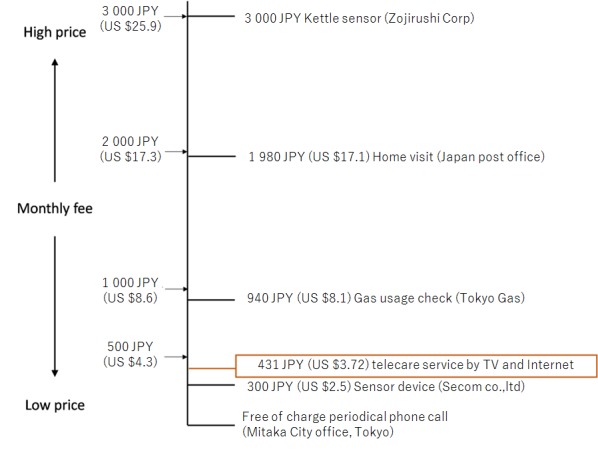
Price comparison with similar services.

According to our research, the estimated median WTP is 431 JPY (US $3.7) per month, and the mean is 809 JPY (US $6.9) per month. It would be greatly beneficial if there were other similar research studies available for comparison. Unfortunately, WTP research for this service is first of its kind, which limited us from comparing with previous research. As the second best alternative, we decided to use figures of similar services to compare the practicality and validity of our research finding. Similar services (Figure 8) include a signaled kettle sensor for 3000 JPY (US $25.9) per month, a periodical home visit service by the post office for 1980 JPY (US $17.1) per month, and an add-on gas meter sensor service for 940 JPY (US $8.1) per month.

Compared with our finding, the prices listed above are more expensive. WTP only represents utility of consumers. However, services provided by privately owned enterprises need to retrieve costs and make a profit on top of the consumer utility. It is worth mentioning that care services are offered by the security company, Secom Ltd, for 300 JPY (US $2.5) per month. Although 300 JPY may seem less costly compared with any other services, this service is provided as an optional service of top of home security services for 5900 JPY (US $51.0) per month. If singled out, the service provided by Secom Ltd is likely to be higher than other services.

There is also a free periodic phone call service provided by Mitaka City in Tokyo but, although it is free of charge, it does not indicate that it is free to run and operate. The only reason this service can exist and continue existing is because of the direct financial support from the government in the form of taxes paid by the general public. In addition to government financial support, Mitaka City does not send any personnel to make home visits, which significantly cuts the cost on human resources.

In conclusion, the WTP figure of 431 JPY (US $3.7) per month is valid. We can make this conclusion because this figure is lower than all privately owned enterprises yet higher than public services provided by Mitaka City.

### Comparing Different Age Groups in WTP

Looking at different age groups, we notice a factor in significant difference in WTP comes from one of the three age groups. *Young generation from 18-39 years old* and *middle generation from 40-64 years old* do not make any significant difference. However, the WTP for *elderly generation (65+ years)* is substantially higher than the other two age groups. In the beginning of this research, we expected the middle age group to have the highest WTP because this group is expected to have the highest income. However, this group is also expected to have the longest working hours, and therefore, it is likely for this age group to pay to ensure the health of their parents. Another reason for this assumption is the marketing direction of privately owned enterprises. If we pay attention to the marketing direction of enterprises offering similar services, we can notice that all targets are set at the middle generation age group. This trend misleads us to think that the middle generation age group would have the highest WTP out of all three age groups. Thus, we misjudged the tendency, and the elderly generation age group has the highest WTP out of all. After this consideration, we believe the reason for the highest WTP for this age group is because of two reasons, the first of which is savings. In this research, we looked at net income but not savings. Although the middle generation age group may have the highest net income, the elderly generation age group has the highest savings out of all three age groups [25], which allows them to spend money on ensuring their own standard of living. The second reason is only an assumption; however, humans are likely to worry about one’s own health risk the most. Therefore, instead of spending money on others, it is more likely that one spends money on themselves, which for this service happens to be the elderly generation age group. As a reflection, we decided that if we were to conduct the same or similar research again, we would add *savings* as a new factor and pay more attention to the *spending pattern* of sampling subjects.

### Comparing Urban and Nonurban Test Subjects in WTP

According to the research done by the Ministry of Internal Affairs and Communications, unnoticed solitary death is higher in urban than in nonurban areas [26]. We believe the reason for this trend is because people living in nonurban areas are not as busy as people living in urban areas, which allows them to have a closer relationship with one another. This close relationship naturally lowers the risk of unnoticed solitary death; therefore, the WTP in nonurban areas, because of direct relationship and closeness with neighbors, should be lower than WTP in urban areas. However, according to our research, the WTP for urban and nonurban does not make much of a difference in WTP. After careful consideration, we believe this is because excessively busy lifestyles in urban areas lower the public consciousness of solitary death in urban areas. Due to the lack of consciousness, the WTP in urban areas is roughly the same as in nonurban areas.

### Analysis of Factor Affecting WTP

During research, we found six different variables that affect the final WTP estimation: *constant factors*, *log-bid*, *gender*, *health consciousness*, *willingness to use*, and *not seen for over a week*. We will look at four out of the six factors. With regard to gender, we expected that, given the same health consciousness, WTP would not change for either gender. However, the result shows that males have higher WTP than females. We believe the reason is because males have higher risks of solitary death compared with females (Tokyo Medical Examiners’ Office). Health consciousness is an easy factor to understand. People with higher health consciousness naturally are more willing to pay to receive health care service and information, thus, their WTP is higher than that of people with lower health consciousness. Our previous study [27] indicated that willingness to use affects the WTP [27].

For the factor see others less than once a week, it is possible that they do not participate in the local society, so they found our project offering information about the local community and telecare service useful.

The technology acceptance model (TAM) is widely used as a model to analyze factors that people accept new technologies and services. In this model, four factors, *perceived usefulness*, *perceived ease of use*, *attitude toward using*, and *behavioral intention to use* are cited as factors leading to the use of new technologies and services by people [28].

Hirose et al are conducting research using TAM for responses to mHealth application on health behavior [29]. From the results, they suggest that it is possible to explain health behavior by TAM. For this service, we also need to consider the technological acceptability by TAM. Furthermore, we think that it is also necessary to consider availability of financial resources and elasticity of pricing to examine the possibility of more realistic service acceptance. We also believe that it is necessary to analyze what kind of causal relationship the new watchkeeping service used in this research can be accepted by using TAM. We will make future investigations to conduct surveys together with analysis by TAM and WTP.

In this research, we were conducting a questionnaire survey in temporary environments; hence, we cannot clarify what factors and performance are affecting the WTP. Analysis of customer satisfaction and physical satisfaction using a service quality model such as the Kano model is needed. On the basis of the analysis results, we believe that it is necessary to consider the acceptance price, taking the quality of service into account.

### Sampling Bias on Internet Research

This research was conducted using an Internet survey; therefore, it is possible that there is bias [30,31]. The biggest bias is information technology (IT) literacy. For people with sufficient IT literacy, it is safe to assume that they are of a healthier group that is not in immediate need of this service, which thus lowers the WTP average. On the other hand, the people who need the service do not have enough IT literacy to answer the survey via the Internet. There is also gender bias to be considered. When we chose the survey sample, we made choices based on age and location, but we did not take gender into consideration, which may lower the accuracy of the final estimated WTP. To prove the existence of gender bias, we made the following table listing percentage of different sample groups (Table 14). The result shows that there is gender inequality in the research sample. If we look at the ratio of male and female in the survey participants, we can see that the male sample group is much greater than the female sample group. The ideal sampling group for male is 193, but the total number of males who took the test was 248, whereas the ideal female sample is 207, but the number of females who took the test was only 152. If we look at these figures using the chi-square test, the number of males in the age group of 50 to 64 years is greater than the expected value, whereas the number of females aged 50 to 64 years and 65+ years is less than the expected value. These numbers indicate that this bias may affect the final estimated WTP. If we were to conduct a similar research next time, it would be important to take the gender difference into account as well.

The double-bound dichotomous choice method used in this study may cause yes-saying bias. It also has the possibility of causing anchoring bias, in which the answer depends on the initial presentation amount. The point that it cannot fully cope with these tasks is considered to be one of the problems of CVM. On the basis of the theory of behavioral economics, we think that it is necessary to consider the WTP in a method not affected by the priming effect and the anchoring effect.

**Table 14:** Chi-square test for gender distribution. χ^2^_20_=104, *P*&lt;.05.

Gender and values		18-29 years	30-39 years	40-49 years	50-64 years	Over 65 years	Total
**Males**							
	Observed value	22	20	41	66	99	248
	Predicted value	29	31	35	45	53	193
	Adjusted residual	−0.55	−0.79	0.49	1.71	4.20	
**Females**							
	Observed value	35	41	28	25	23	152
	Predicted value	28	30	34	46	70	207
	Adjusted residual	0.67	1.09	−0.59	−2.20	−5.48	

### Conclusions

In this study, we conducted an Internet survey. Median WTP is estimated to be 431 JPY (US $3.7) per month, and mean WTP is estimated to be 809 JPY (US $6.9) per month. For comparison of WTP among age groups and differences between residential areas, the elderly group is estimated to be higher than other groups. In the analysis of factors that affect WTP, *constant factors*, *log-bid*, *gender*, *health consciousness*, *willingness to use*, and *not seen for over a week* had positive effects on WTP. With regard to *gender*, it was estimated that there was a significant effect only for men. In comparison of WTP in different groups, age groups showed that WTP of the elderly group was higher than WTP of the middle age group and younger age group. However, WTP surveys need to be carefully conducted to minimize the sampling bias and to allocate accurate structure of gender distribution.

## Abbreviations

CVM: contingent valuation method
IT: information technology
VIF: variance inflation factor
WTP: willingness to pay
